# TWEAK-Fn14: a promising target for cardio-cerebrovascular diseases and brain-heart syndrome

**DOI:** 10.3389/fimmu.2025.1710055

**Published:** 2026-04-01

**Authors:** Qinglin Fei, Jun Chang, Tingcan Jiang, Guoqiang Guan, Yujuan Liang, Cuicui Cheng, Guangxu Xiao, Qiuyan Guo, Guanwei Fan, Yan Zhu, Ming Lyu

**Affiliations:** 1 State Key Laboratory of Component-Based Chinese Medicine, Tianjin University of Traditional Chinese Medicine, Tianjin, China; 2 State Key Laboratory of Chinese Medicine Modernization, Tianjin University of Traditional Chinese Medicine, Tianjin, China; 3 Departement of Neurology, Jiaxing Hospital of Traditional Chinese Medicine, Jiaxing, Zhejiang, China; 4 State Key Laboratory for Quality Ensurance and Sustainable Use of Dao-di Herbs, Artemisinin Research Center, and Institute of Chinese Materia Medica, China Academy of Chinese Medical Sciences, Beijing, China; 5 National Clinical Research Center for Chinese Medicine Acupuncture and Moxibustion, First Teaching Hospital of Tianjin University of Traditional Chinese Medicine, Tianjin, China

**Keywords:** TWEAK-Fn14, cardiovascular diseases, cerebrovascular diseases, brain-heart syndrome, immuno-inflammation

## Abstract

Cardio-cerebrovascular diseases (CCVDs), notably stroke and coronary heart disease, represent the leading causes of mortality and disability worldwide. The intricate bidirectional feedback between the brain and heart in brain-heart syndrome (BHS) exacerbates clinical outcomes and imposes a significant economic burden on patients. The cytokine tumor necrosis factor-like apoptosis weak inducer (TWEAK) and its receptor fibroblast growth factor-inducible 14 (Fn14) are overexpressed in cerebral injury and cardiac dysfunction. Elevated levels of TWEAK and Fn14 contribute to the development of various brain diseases, including blood-brain barrier damage, brain edema, neuroinflammation, neuronal apoptosis, and neurodegeneration. Additionally, the TWEAK-Fn14 axis is implicated in numerous pathophysiological events in the heart, such as cardiomyocyte proliferation, inflammation, apoptosis, hypertrophy, fibrosis, contractile function disruption, and ventricular dilatation. Given its significant contributions to CCVDs, the TWEAK-Fn14 axis has also emerged as a promising therapeutic target for BHS. In this review, the critical roles of TWEAK-Fn14 in CCVDs and its potential interplay between the brain-heart axis in BHS were updated and discussed, which shed a new light on co-treatment of brain and heart and brain-heart syndrome.

## 1 Introduction

Cardio-cerebrovascular diseases (CCVDs), primarily comprising heart disease and stroke, are the leading cause of mortality, accounting for approximately 17 million deaths annually, a number that is projected to continue increasing (1). The pathophysiological basis of cardiovascular diseases (CVDs) is vascular and cardiac remodeling, resulting from the myocardium and vascular response to a range of hemodynamic, metabolic, and inflammatory stimuli. Stroke, classified as one of the cerebrovascular disorders, exerts severe impacts on brain function. Stroke can be categorized into two primary types: ischemic and hemorrhagic (2). Ischemic stroke accounts for approximately 85% of all stroke cases, while hemorrhagic stroke constitutes the remaining 15% (3–5). The underlying causes of these two stroke types are distinct. Ischemic stroke occurs due to a lack of blood flow to the brain, often caused by hypoxia. Conversely, hemorrhagic stroke results from the rupture or leakage of intracranial blood vessels. The physiological impact of stroke can be profound, triggering a range of pathophysiological cascade responses, which include disturbances in energy metabolism, unregulated release of excitatory neurotransmitters, depolarization of neurons and glial cells, damage to the blood-brain barrier (BBB), elevated intracellular calcium levels, glial cell activation, inflammation, and ultimately, apoptotic cell death. These changes have lasting effects on brain health and function (6–10).

Over the past decade, a growing body of evidence from clinical trials, animal studies, and neuroimaging have emerged, highlighting the prevalence of cardiac dysfunction following stroke in clinical practice (11). Specifically, clinical trials have revealed that nearly 20% of patients with ischemic stroke experience severe adverse cardiac events, such as arrhythmias, myocardial ischemia, and acute coronary syndrome, within three days (12). Furthermore, Brain-heart syndrome (BHS) is triggered by stroke, encephalopathy, or traumatic brain injury, particularly affecting the hypothalamus, brainstem, and autonomic nervous system (13, 14). This syndrome often leads to complications like acute myocardial infarction (AMI), myocardial ischemia, cardiac arrhythmia, stress cardiomyopathy, and even HF, further emphasizing the intricate relationship between the heart and the brain (15, 16). BHS has emerged as a frontier research area in recent years, attracting widespread attention from esteemed global experts. According to various authoritative reviews, the potential mechanisms underlying stroke-induced BHS have been systematically explored, encompassing the hypothalamic-pituitary-adrenal (HPA) axis system, microbiota regulation, inflammatory and immune responses, and microvesicles (11, 14, 16, 17). Notably, studies have revealed that the β2-adrenoceptor signaling pathway significantly influences the intricate brain-cardiac-renal network (18). Additionally, the β-blocker metoprolol has demonstrated efficacy in mitigating chronic cardiac systolic dysfunction triggered by heightened sympathetic activity stemming from focal cerebral ischemia in mice (19). Furthermore, following an ischemic stroke in rats, the brain can send apoptosis, necrosis, and autophagy signals to cardiac myocytes (20), highlighting the profound interconnection between the brain and heart following stroke. In terms of inflammatory and immune regulation, a seminal study identified IL-1β as a critical driver of pro-inflammatory monocyte infiltration and subsequent cardiac fibrosis following stroke (21). In addition, gut microbiome-derived indole-3-propionic acid attenuates GPX4-mediated ferroptosis, thereby ameliorating myocardial injury in the context of depression via the brain-gut-heart axis (21). Furthermore, recent evidence demonstrates that vagal stimulation exerts cardioprotective effects by suppressing Ccrl2^+^ macrophages through the α7nAChR-NRF2 pathway, suggesting a novel brain-heart cross-organ mechanism for preventing heart failure (22). Despite the clinical significance of the functional and structural changes in the heart following stroke, there remains a scarcity of drugs that specifically target the brain-heart axis for the precise treatment of BHS (14). Therefore, it is of utmost importance to delve deeper into the therapeutic agents and targets for stroke-induced BHS. Notably, tumor necrosis factor (TNF) superfamily proteins have been shown to play a pivotal role in the progression of CCVDs.

Tumor necrosis factor-like apoptosis-inducing factor (TWEAK) and its sole functional receptor, fibroblast growth factor-inducible factor 14 (Fn14) are two members of the TNF superfamily. This review aims to provide a comprehensive update on the TWEAK/Fn14 pathway, encompassing its structure, sources, signaling, functions, and therapeutic targeting in the context of CCVDs. Furthermore, we summarize and discuss its potential mechanisms and roles in BHS.

## 2 TWEAK and its functional receptor Fn14

### 2.1 Structure, origin, expression of TWEAK and Fn14

**Figure 1:**
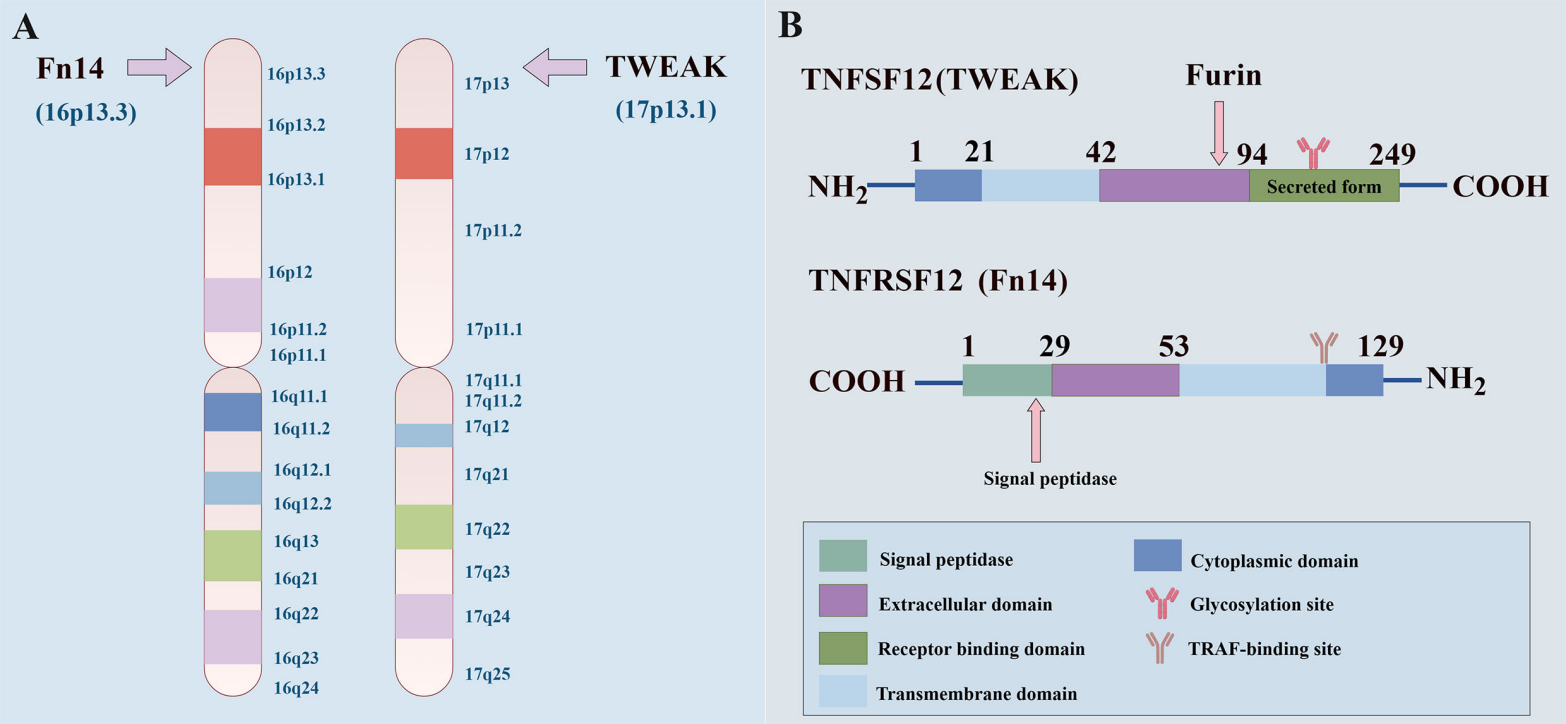
The structure TWEAK and Fn14. **(A)** The human TWEAK and Fn14 genes occupy distinct locations on chromosomes 17p13.1 and 16p13.3, respectively. **(B)** TWEAK, a 249-amino acid type II transmembrane glycoprotein, undergoes proteolytic cleavage by furin protease, releasing a soluble, biologically active 156-amino acid cytokine. Meanwhile, Fn14, a 129-amino acid type I transmembrane protein, undergoes processing by signal peptidases to yield the mature 102-amino acid receptor.

TWEAK was initially discovered in a macrophage cDNA library (23) and was recognized as a member of the tumor necrosis factor superfamily (TNFSF) in 1997 (24, 25). However, in 1998, the TWEAK receptor was incorrectly labelled death receptor 3 (DR3) (26). As the twelfth member of the TNFSF, TWEAK is a type II transmembrane ligand. It has an intracellular N-terminus and an extracellular C-terminus. A hydrophobic transmembrane domain anchors the protein in the membrane, while the C-terminal extracellular domain contains the receptor binding site (27). Located on chromosome 17p13.1, the TWEAK gene encodes a protein consisting of 249 amino acids with a molecular weight of 30 kDa. This protein undergoes hydrolytic processing by furin in the trans-Golgi network, forming a smaller, soluble version of TWEAK (sTWEAK) with 156 amino acids. This soluble form exerts its effects on distal tissues via the systemic circulation (23, 28). Amino acid residues 90–93 have been pinpointed as the primary recognition site for furin during this proteolytic process (29) (**Figure 1**).

In 2001, the receptor for TWEAK was successfully cloned and designated as the multifunctional receptor Fn14. It belongs to the tumor necrosis factor receptor superfamily member 12A (TNFRSF12A) and is commonly known as CD266, having been identified from a cDNA expression library (28, 30, 31). The Fn14 gene on chromosome 16p13.3 is the smallest molecular weight member within the TNF receptor superfamily, encoding a 129 amino acid type I membrane protein. Notably, Fn14 harbors a 27-aa N-terminal signal peptide sequence that undergoes cleavage by signal peptidases, ultimately resulting in a 102-amino acid cell surface receptor (30, 32) (**Figure 1**). Despite its short cytoplasmic tail, comprising only 28 amino acid residues and lacking a death domain, Fn14 possesses a significant binding domain for the tumor necrosis factor receptor-associated factor (TRAF). TRAF is an essential class of adapter molecules. Upon phosphorylation, TRAF binds to Fn14, initiating TWEAK signaling, encompassing TRAF1, TRAF2, TRAF3, and TRAF5. Notably, TRAF2 can form a complex with the cellular inhibitor of apoptosis protein 1 (cIAP1), known as the cIAP1-TRAF2 complex, which is recruited following the activation of Fn14 (33, 34).

Most TWEAK mRNA is expressed predominantly by innate immune cells in the peripheral blood, including polymorphonuclear leukocytes, macrophages, dendritic cells (DCs), natural killer (NK) cells, and subsets of B and T cells (35, 36). Furthermore, TWEAK mRNA has also been detected in endothelial and astrocyte cells. Interestingly, Fn14, on the other hand, is not expressed on the surface of T or B lymphocytes but is localized to the surface of monocytes, macrophages, progenitors, cardiac fibroblasts, endothelial cells, and other non-hematopoietic cells.

In 1999, Fn14 was identified as an immediate-early response gene induced by fibroblast growth factor 1 (FGF1) in mouse NIH3T3 fibroblasts (31). Similarly, in neonatal rat cardiomyocytes, the expression of Fn14 can be stimulated by various factors, including fibroblast growth factor 1 (FGF-1), phenylephrine (PE), angiotensin II (Ang II), and endothelin-1 (ET-1), a process mediated by the RhoA/ROCK pathway (37). TWEAK is widely expressed in many organs and tissues under pathological conditions, such as the heart, intestine, lung, brain, skeletal muscle, and blood vessels, while its expression is low in healthy tissues (7, 23, 38). In contrast to its low or undetectable expression in healthy tissues various pathological conditions can rapidly and substantially upregulate Fn14 expression (39–41).

### 2.2 TWEAK-Fn14 axis: upstream regulators and downstream signaling pathways

**Figure 2:**
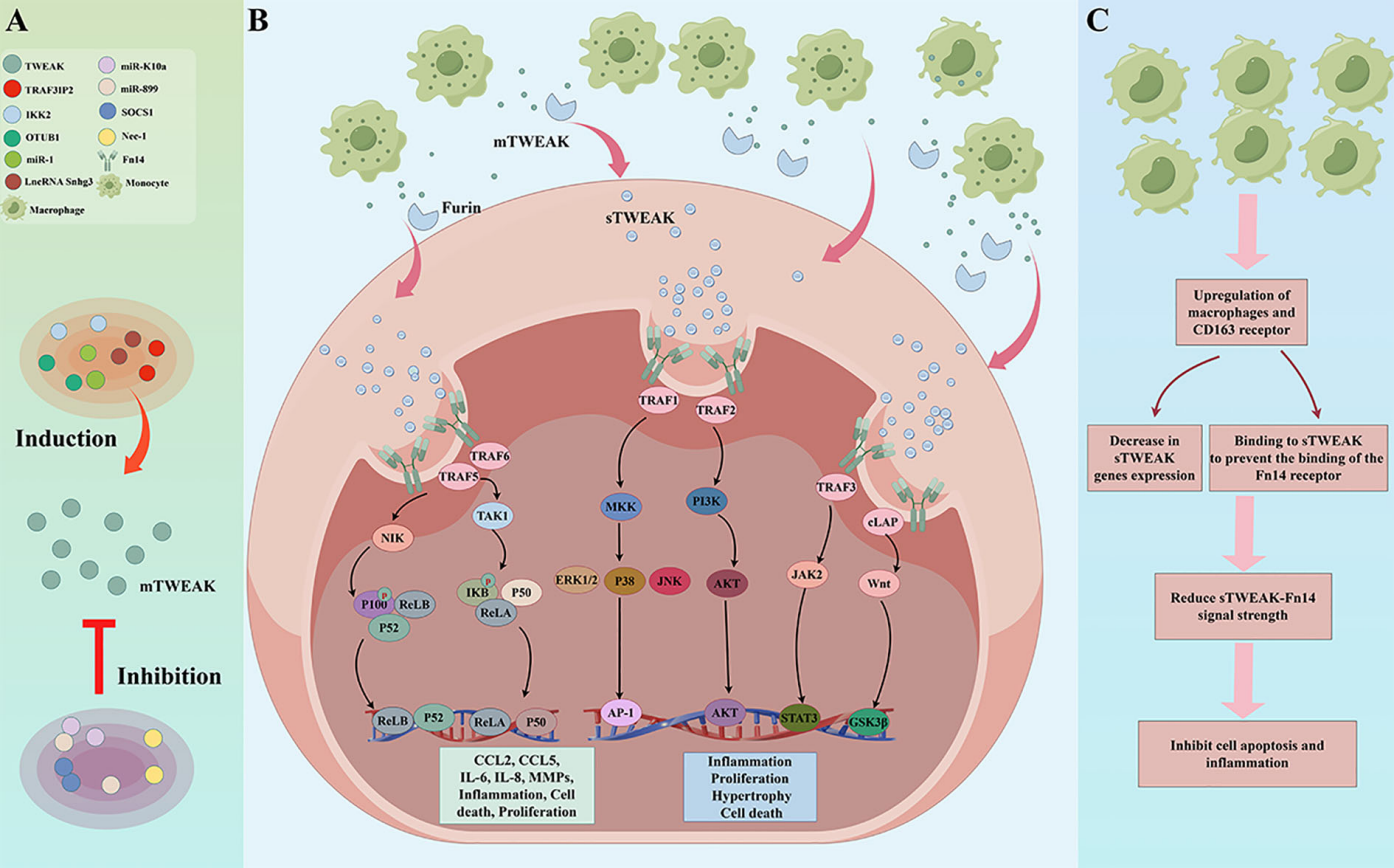
The upstream regulators and downstream signalling pathways of TWEAK-Fn14 affect various cell functions. **(A)** Regulators (including microRNAs, lncRNA, and transcription factors) mediate the TWEAK-Fn14 interaction, inducing or inhibiting TWEAK expression. **(B)** TWEAK-Fn14 binding recruits TRAF proteins, activating signalling pathways (PI3K-AKT, JAK2-STAT3, Wnt-GSK3β) to induce cell inflammation, proliferation, death, and hypertrophy. **(C)** In monocytes and macrophages, sTWEAK binding to CD163 receptor leads to its internalisation, inhibiting downstream signalling and thereby suppressing apoptosis and inflammatory responses.

Upstream regulators modulate the cellular functions of the TWEAK/Fn14 axis and the activation of its downstream signaling pathways. These regulators include microRNAs (e.g., miR-K10a and miR-1), the transcription factor IκB kinase 2 (IKK2), the long noncoding RNA Snhg3, and proteins such as TRAF3IP2, necrostatin-1 (Nec-1), OTUB1, and SOCS1 (42–49) (**Figure 2A**).

By binding to the extracellular structural domain of Fn14, TWEAK initiates receptor trimerization, subsequently activating numerous signaling pathways (50) (**Figure 2B****).** As shown in **Figure 1**, Fn14 is a small Type I transmembrane protein, and its signal transduction activity is primarily concentrated in its short cytoplasmic tail. This trimeric structure, facilitated by its TRAF-binding motif (PIEET), effectively recruits adaptor proteins such as TRAF2 and TRAF5 to the cytoplasmic tail of Fn14 (the region central to Fn14 signaling, whose structure is detailed in **Figure 1**), fostering functional interactions between them and thus activating diverse signaling cascades (35). Furthermore, Fn14 can activate the NF-κB and mitogen-activated protein kinase (MAPK) pathways, leveraging the PIEET binding motif (33, 51, 52). The PIEET-adjacent KFTT sequence, as part of this critical intracellular signaling domain, emerges as a vital component in this process. Remarkably, the deletion of KFTT led to a complete abrogation of NF-κB activation by Fn14, whereas PIEET did not exhibit a comparable impact on NF-κB activation (35).

The binding of TWEAK to Fn14 facilitates Fn14 trimerization, significantly intensifying the recruitment of TNF receptor-associated factors (TRAF) to initiate downstream signaling pathways, including the classical and non-classical NF-κB pathways (51, 53), as well as the PI3K/AKT, Wnt/GSK3β (38, 54–59), JAK2/STAT3 (47, 60), and transforming growth factor-[deta]-activated kinase-1 (TAK1) (55) pathways. Moreover, the TWEAK/Fn14 complex triggers phosphorylation of the downstream mitogen-activated protein kinase (MAPK) pathway (52, 55, 61, 62), encompassing three major subpathways: the extracellular regulated protein kinase (ERK), c-Jun amino-terminal kinase (JNK), and p38 protein kinase. The potency of TWEAK signaling is bolstered by the activation of the Wnt signaling pathway and the dephosphorylation of GSK3β (56, 63, 64). TWEAK is synthesized as the full-length, membrane-bound form (mTWEAK). It is then rapidly and constitutively cleaved by the serine endoprotease furin, a process that is often upregulated in inflammatory conditions, to yield the mature soluble fragment (sTWEAK) (29, 65). Both sTWEAK (circulating and distant acting) and mTWEAK (contact-dependent and local acting) can bind to Fn14. This dual-form nature means that when TWEAK is discussed broadly, it refers to the combined activity of both forms; however, studies focusing on systemic biomarkers primarily measure sTWEAK levels in circulation (65). Importantly, the type of TWEAK (membrane or soluble) can influence the downstream NF-κB activation profile; specifically, mTWEAK is often associated with predominant canonical NF-κB activation, while sTWEAK preferentially promotes non-canonical NF-κB signaling, though this varies by cell type (65). Furthermore, both membrane-bound and soluble TWEAK play pivotal roles in diverse physiological and pathological conditions *in vivo (*29).

Cluster of Differentiation 163 (CD163) is a cysteine-rich scavenger receptor superfamily member with a molecular weight of 130 kDa. It is currently considered a single-pass transmembrane glycoprotein located exclusively on the plasma membrane of monocyte-derived macrophages. CD163, a hemoglobin-scavenging receptor, was identified in 2007 as another receptor for TWEAK (66, 67). TWEAK has been shown to be internalized upon binding to CD163, suggesting that CD163 functions as a scavenger receptor (66, 68, 69). Moreover, regarding biological responses, CD163 mitigates the deleterious biological effect of TWEAK (70). It is well known that TWEAK interacts with CD163 and plays a crucial role in tissue repair after ischemic injury, but more research is needed to explore the role of the TWEAK/CD163 interaction in disease in the future (71) (**Figure 2C**).

The interaction between TWEAK and Fn14 triggers a diverse array of pathophysiological cellular functions, with the resulting effects contingent upon the specific cell type and microenvironment through these signaling pathways. TWEAK has emerged as a key player in numerous cellular and biological functional processes, encompassing pro-inflammatory responses (54, 72–74), cell proliferation (53, 75), migration (76, 77), apoptosis (78, 79), and differentiation (80, 81).

## 3 TWEAK-Fn14 in cardio-cerebrovascular diseases

**Figure 3:**
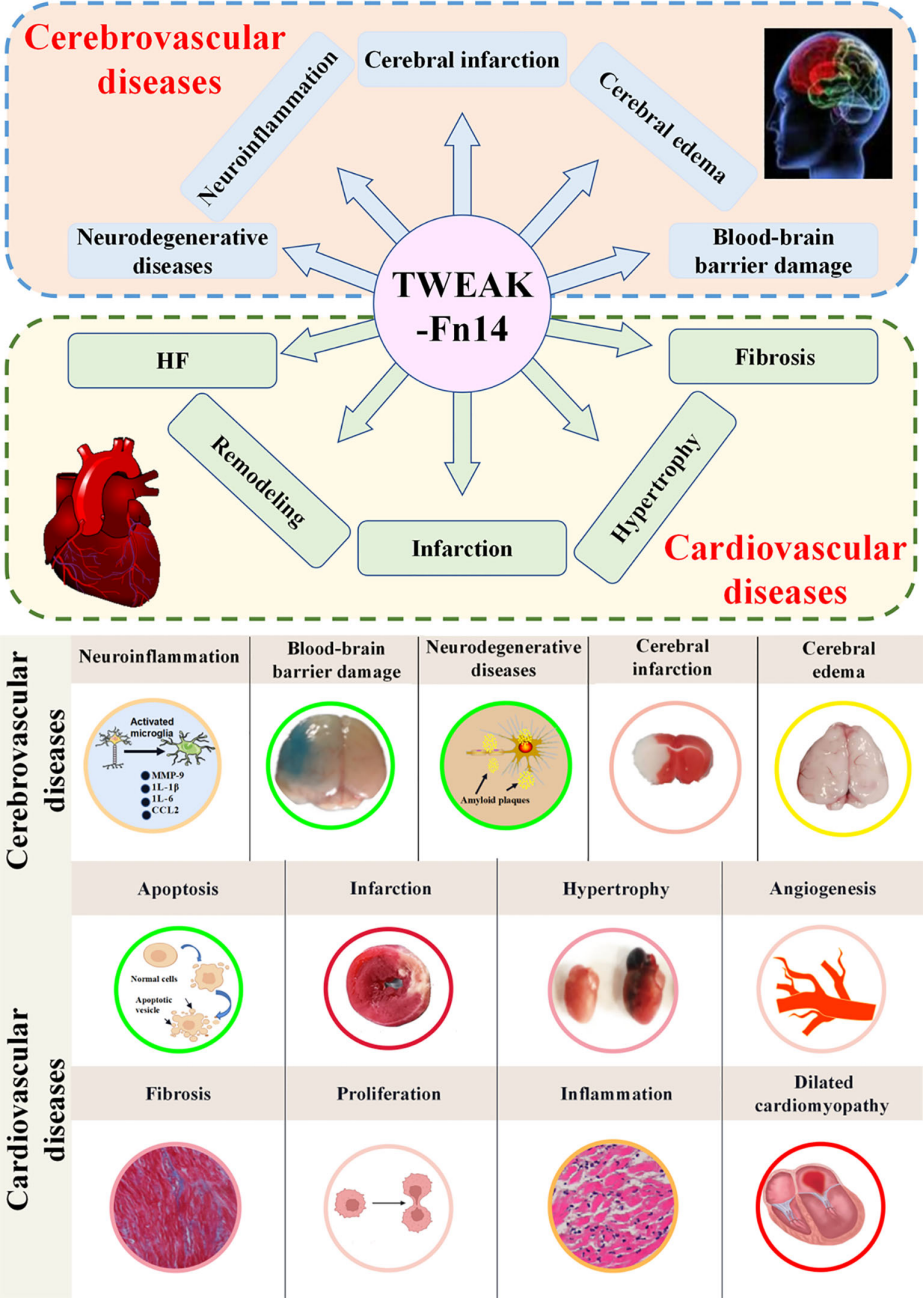
The functions of TWEAK-Fn14 in cardio-cerebrovascular diseases. High levels of TWEAK are intricately linked to a broad spectrum of brain and heart diseases. When brain injury occurs, it diminishes blood flow to the brain, subsequently igniting a complex chain reaction in ischemic neurons. This cascade encompasses damage to the blood-brain barrier, cerebral edema, neurodegeneration, neuroinflammation, and neuronal apoptosis. As this progression unfolds, a series of reactions activate microglia, spurring the production of pro-inflammatory cytokines, chemokines, and matrix metalloproteinases (MMPs). Moreover, brain injury can foster the development of β-amyloid plaques, which are extracellular protein deposits often associated with neurodegenerative disorders. On the cardiac front, TWEAK-related diseases encompass angiogenesis, cardiomyocyte proliferation, hypertrophy, necrosis, apoptosis, and interstitial fibrosis, all of which provoke inflammatory responses. These pathological alterations in the heart have the potential to culminate in dilated cardiomyopathy, ultimately leading to HF.

TWEAK and its cognate receptor Fn14 occupy pivotal positions in the pathogenesis of CCVDs. Elevated levels of TWEAK trigger a diverse array of brain-related pathologies, such as BBB impairment, which promotes brain edema, neurodegeneration, neuroinflammation, and neuronal apoptosis. Furthermore, this upregulation is associated with a spectrum of cardiac pathological alterations, including cardiomyocyte proliferation, hypertrophy, necrosis, apoptosis, inflammation, interstitial fibrosis, and systolic dysfunction, as depicted in **Figure 3**. These findings underscore the significant impact of TWEAK-Fn14 signaling on CCVDs.

### 3.1 TWEAK-Fn14 in cerebrovascular diseases

#### 3.1.1 TWEAK-Fn14 in stroke

**Figure 4:**
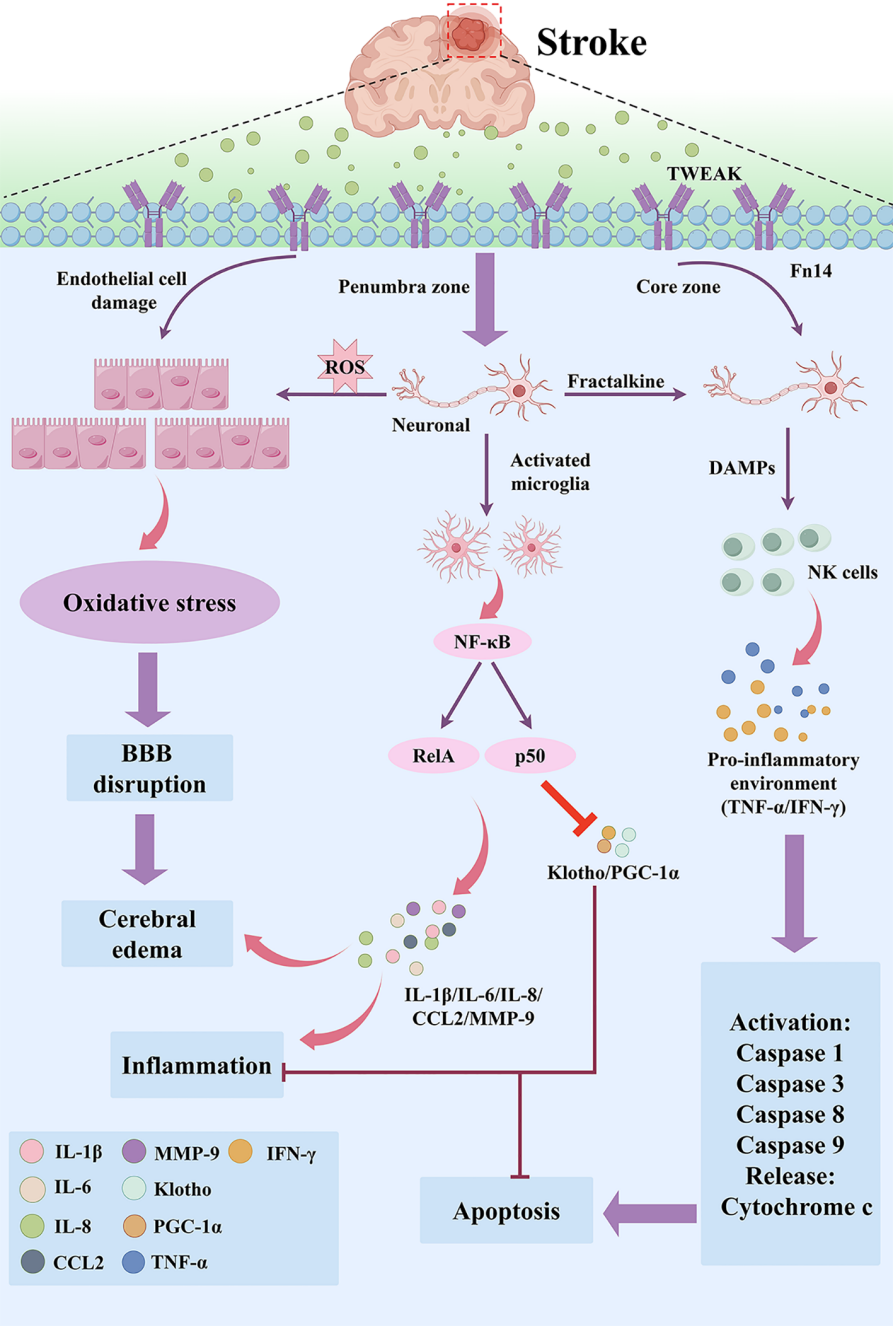
Key mechanisms of TWEAK–Fn14 signaling in stroke pathogenesis. Cerebral ischemia activates TWEAK–Fn14 signaling, promoting neuronal apoptosis, neuroinflammation, and oxidative stress. Microglial activation triggers the NF-κB pathway, upregulating proinflammatory mediators (e.g., IL-1β, IL-6, CCL2, MMP-9) and downregulating protective proteins (Klotho, PGC-1α). Increased ROS in the penumbra exacerbates oxidative stress, disrupting the blood–brain barrier (BBB) and inducing cerebral edema. In the ischemic core, necrosis releases DAMPs, recruiting NK cells that secrete IFN-γ and TNF-α. These cytokines enhance caspase activation (caspases 1, 3, 8, 9), cytochrome C release, and apoptotic vesicle formation, perpetuating inflammation and apoptosis and worsening neurological injury.

TWEAK plays a pivotal role in the delayed phase of ischemic brain injury, encompassing phenomena such as neuronal apoptosis and neuroinflammation in the penumbra and brain edema. This role potentially extends the window for therapeutic intervention in brain injury (82, 83). Additionally, in the pathogenesis of stroke, the TWEAK-Fn14 axis is intricately implicated in neuronal apoptosis and disruption of the BBB (66, 82–85) (**Figure 4****).** *In vitro* experiments on cortical neurons using an oxygen-glucose deprivation/reoxygenation (OGD/R) model have demonstrated upregulation of TWEAK and Fn14 gene expression levels within 3 to 6 hours (84). However, *in vivo* following MCAO, an increase in Fn14 mRNA level was detected within 24–48 hours, with peak expression observed at 48 hours, and the level remained above baseline at 72 hours (86). Similarly, when examining the cerebral cortex after 24 hours of middle cerebral artery occlusion (MCAO), the Fn14 mRNA level was upregulated by a factor of 22.3 (84). These findings align with the adverse effects of the TWEAK-Fn14 axis on cerebral ischemia. Crucially, the TWEAK-Fn14 axis exerts its detrimental effects by actively suppressing two crucial neuroprotective factors: α-Klotho protein (Klotho) and peroxisome proliferator-activated receptor-γ coactivator-1α (PGC-1α). This suppression is directly linked to the inflammatory signaling cascade initiated by the TWEAK-Fn14 interaction. Upon activation, TWEAK-Fn14 strongly activates the pro-inflammatory NF-κB pathway, and it is this robust NF-κB signal that serves as a key negative regulator, suppressing the expression of both Klotho and PGC-1α. Klotho, a neuroprotective protein, normally protects against neural injury through its antioxidant properties (87, 88). However, TWEAK-induced NF-κB activation is confirmed to negatively regulate the expression of the Klotho gene (89), causing a reduction in its protective capacity against oxidative stress and inflammation, thereby exacerbating tissue injury. Similarly, PGC-1α is fundamentally essential for mitochondrial metabolism, biogenesis, and homeostasis, particularly during acute stroke when overproduction of reactive oxygen species (ROS) leads to mitochondrial dysfunction (90–93). PGC-1α maintains neuroprotection via its downstream targets, UCP2 and SOD2, which mitigate ROS release (94–98). By activating NF-κB, the TWEAK-Fn14 axis functions as a strong inhibitor of PGC-1α expression and activity (99). This inhibition directly undermines mitochondrial biogenesis and function, amplifying ROS production and contributing to delayed neuronal death. In essence, by actively downregulating these protective pathways, TWEAK-Fn14 amplifies both the acute and delayed phases of ischemic injury, underscoring the potential efficacy of targeting the TWEAK-Fn14 axis as a therapeutic strategy for cerebral ischemia (84).

##### 3.1.1.1 TWEAK-Fn14 in neurovascular unit

The neurovascular unit (NVU) encompasses the structural and functional components of the BBB, a dynamic ensemble of cellular, stromal, and neuronal structures. This intricate network plays a pivotal role in maintaining cerebral homeostasis, predominantly composed of capillary endothelial cells, neurons, astrocytes, microglia, and the extracellular matrix. It was revealed that TWEAK, a critical biological factor, exerts harmful effects on the NVU’s structure and the BBB’s permeability. Furthermore, the permeability of the NVU exhibits a dose-dependent relationship with recombinant TWEAK (100). Specifically, the direct administration of recombinant TWEAK into the brain prompts the activation of NF-κB and the subsequent MMP-9 expression, which leads to a surge in BBB permeability (85, 100, 101). Intriguingly, following the intracerebral injection of recombinant TWEAK in p50 gene-deficient animals (p50 is a core component of the NF-κB canonical activation dimer), neither disruption of the BBB nor phenotypic manifestations of brain edema were observed. This suggests that the TWEAK-induced activation of the NF-κB pathway is a pivotal factor in increasing BBB permeability compromising its integrity (100). Moreover, the injection of TWEAK was found to not only induce perivascular astrocyte edema but also cause the detachment of astrocyte peduncles from the basement membrane, ultimately leading to the formation of a perivascular edema zone (100). Remarkably, Fn14 fusion proteins and Fn14 gene defects have been shown to inhibit MMP-9 activation induced by cerebral ischemia, effectively reducing NVU permeability (100). Moreover, treatment with Fn14-Fc decoy inhibits cerebral ischemia-induced degradation of laminin in the basal lamina (85). In agreement with these findings, the genetic or pharmacological inhibition of the TWEAK/Fn14 axis reinforces the integrity of the BBB in various models. For example, Fn14-TRAIL-expressing mice (a decoy receptor combining Fn14 and TNF-related apoptosis-inducing ligand) with experimental autoimmune encephalomyelitis showed enhanced BBB integrity in the spinal cord, brainstem, and cerebellum (102). Similarly, Knocking out Fn14 in MRL/lpr mice (a widely used murine model of systemic lupus erythematosus and lupus nephritis) can reduce IgG deposition in brain tissue, thereby lowering BBB permeability (103).

##### 3.1.1.2 TWEAK-Fn14 in neuronal apoptosis and neuroinflammation

Apoptosis, a key pathogenic factor in stroke, is synonymous with programmed cell death. Under ischemic conditions, the outer mitochondrial membrane liberates Cytochrome c (Cyt-c), triggering the formation of apoptotic bodies that activate caspase-9 and caspase-3, resulting in the cleavage of the essential enzyme poly (ADP-ribose) polymerase-1 (PARP-1) (104) in the intrinsic apoptotic pathway. TWEAK-Fn14 signaling significantly contributes to this injury by acting as a potent pro-apoptotic mediator. Recent studies have demonstrated that under oxygen-glucose deprivation/reperfusion (OGD/R) conditions, TWEAK prompts neuronal cell death by activating the NF-κB pathway, thereby enhancing inflammatory factor production and contributing to the activation of caspase-3 (105). *In vivo*, the axis activates transcription factor NF-κB through the upstream kinase Iκκ [IκαppαB (inhibitory κappaB) kinase], promoting neuronal cell death. Inhibition of this NF-κB activation, such as employing a dominant-negative Iκκ form via a recombinant adenovirus or pharmacological inhibition of Iκκ activity, effectively reduced the extent of cerebral ischemic infarction and neuronal cell loss (85, 105, 106). Beyond direct effects on neuronal viability, TWEAK-Fn14 drives broader neuroinflammation through the activation of microglia. Microglia are the central nervous system’s resident macrophages and possess dual roles (neuroprotective vs. neurotoxic). However, in the context of TWEAK signaling, NF-κB activation within these microglia primarily promotes a pro-inflammatory phenotype (M1 polarization) that is strongly linked to secondary neuronal degeneration (105, 107–109). This neurotoxic activation, which TWEAK elicits for the canonical NF-κB pathway both *in vitro* and *in vivo* (100), exacerbates ischemic injury through indirect inflammatory mechanisms. Drawing from these insights, the inhibition of the TWEAK-Fn14 axis (such as through Fn14-Fc fusion proteins or Fn14 gene defects which inhibit the phosphorylation of Iκκβ and IκBα following MCAO (85)) presents a compelling strategy to mitigate both direct neuronal apoptosis and indirect neuroinflammatory damage in cerebral ischemia.

### 3.2 TWEAK-Fn14 in the CVDs

**Figure 5:**
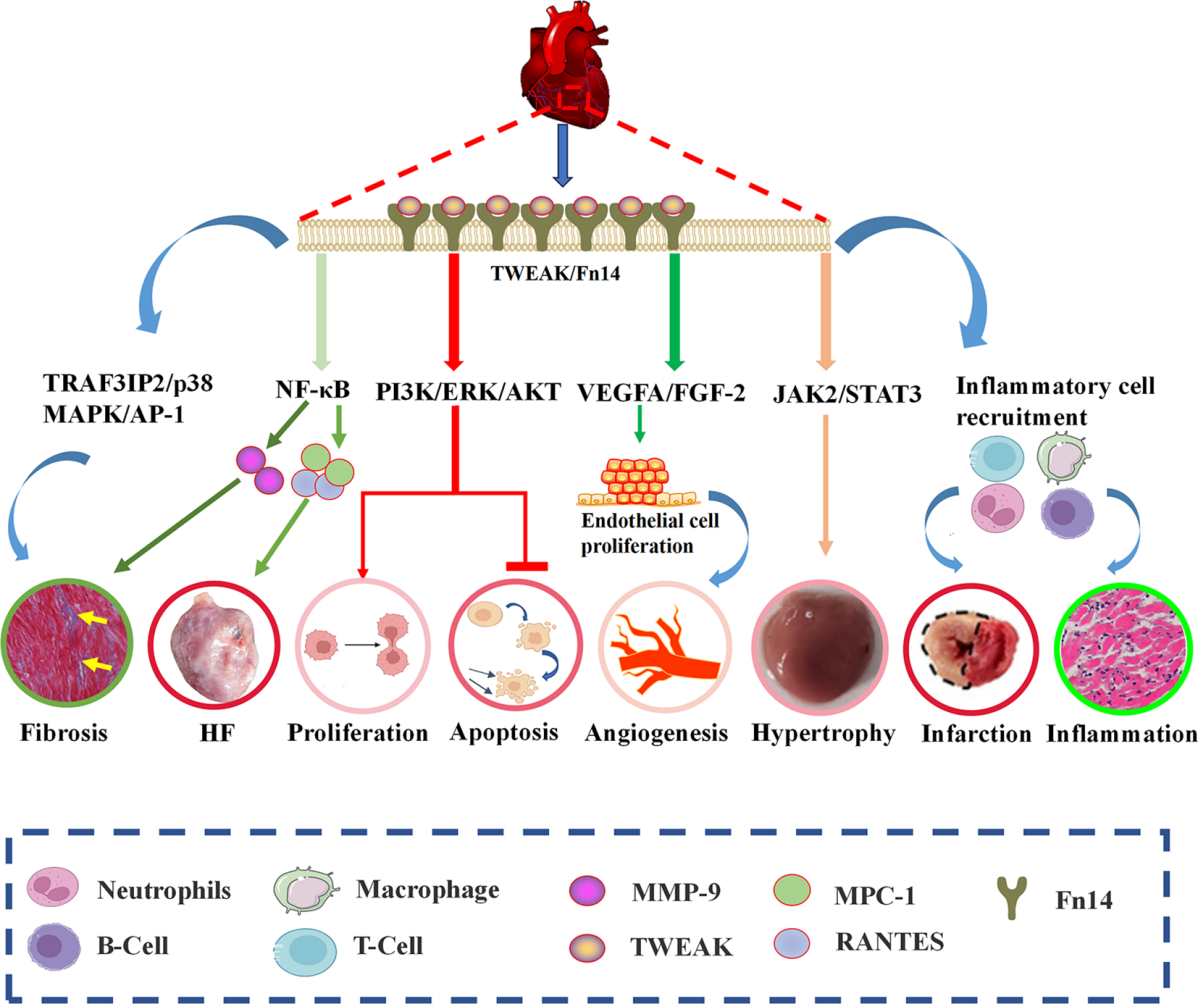
The TWEAK-Fn14 pathway controls multiple cellular activities and is involved in multiple biological functions associated with CVDs. TWEAK, upon binding to the Fn14 receptor, effectively stimulates cellular proliferation and inhibits apoptosis by activating the PI3K/ERK/AKT signaling cascade. TWEAK-Fn14 complex significantly enhances the vascular endothelial growth factor A (VEGFA)- and fibroblast growth factor-2 (FGF-2)-mediated proliferation and angiogenesis in human endothelial cells. Furthermore, the activation of TWEAK-Fn14 triggers cardiomyocyte hypertrophy via the JAK2/STAT3 signaling pathway. Additionally, TWEAK elevates TRAF3IP2 expression and promotes its nuclear translocation, thereby inducing the activation of p38 MAPK, NF-κB, and AP-1, accompanied by collagen secretion and deposition, ultimately leading to cardiac fibrosis. Specifically, the activation of the NF-κB signaling pathway results in the synthesis of MMP-9 and an increased expression of proinflammatory factors such as MCP-1 and RANTES, which significantly contribute to cardiac fibrosis and HF. Moreover, the activation of the TWEAK-Fn14 signaling pathway also triggers the infiltration of inflammatory cells, including macrophages, B cells, T cells, and neutrophils, ultimately resulting in myocardial inflammation and myocardial infarction. This process further underscores the intricate role of TWEAK-Fn14 signaling in various cardiovascular pathologies.

CVDs frequently manifest not only in structural alterations within the heart but also in functional changes. Among the myriad pathological events associated with this condition are cardiac hypertrophy, fibrosis, remodeling, and inflammation. Moreover, the progression of CVDs is often influenced by the intricate interplay between members of the TNFSF and their respective receptors (110). TWEAK, a vital component of the TNFSF, functions as a pro-inflammatory and pro-angiogenic cytokine, playing a crucial role in physiological tissue regeneration and repair. In addition, TWEAK is strongly correlated with cardiac remodeling, dysfunction, myocardial fibrosis, myocardial infarction, and cardiomyocyte apoptosis, as depicted in **Figure 5**.

#### 3.2.1 TWEAK-Fn14 in cardiomyocyte proliferation

The proliferation of cardiomyocytes plays a pivotal role in the growth of the heart during embryonic development. Nevertheless, human cardiomyocytes undergo a notable decline in their differentiation and proliferation abilities shortly after birth, often within the first week (111). While the impact of the weakly apoptosis-inducing cytokine TWEAK on cardiomyocyte proliferation has been documented, its precise mechanism remains elusive (56). TWEAK can significantly induce the expression of regulators that are crucial for cardiomyocyte proliferation in neonatal mice. This occurs through its binding to Fn14, activating downstream signaling pathways of ERK and PI3K/AKT. Fn14 overexpression potently augments the likelihood of TWEAK-induced DNA synthesis in adult cardiomyocytes. During embryonic development, Fn14 expression is elevated and shows a positive correlation with the cardiomyocyte proliferation rate. As cardiomyocytes mature, this proliferation rate gradually declines (56). However, current research indicates that the TWEAK-Fn14 axis is highly responsive to cell cycle re-entry and DNA replication in adult cardiomyocytes, positioning the axis as a potentially potent inducer of cardiomyocyte proliferation. Intriguingly, TWEAK not only stimulates endogenous neonatal rat cardiomyocytes to induce Fn14 expression but also directly triggers cardiomyocyte proliferation, underscoring its potential therapeutic value in cardiac regeneration.

#### 3.2.2 TWEAK-Fn14 in cardiomyocyte apoptosis

A pivotal determinant in the cardiac recovery process following AMI is cardiomyocyte apoptosis. The research shows that drug therapy and genetic disruption techniques can effectively hinder cardiomyocyte apoptosis, thereby promoting myocardial repair (112, 113). Importantly, the role of the TWEAK/Fn14 signaling pathway is highly dependent on the specific context and phase. In contrast to its primarily detrimental role in cerebral ischemia, the TWEAK-Fn14 signaling in the heart exhibits a phase-dependent, meaning it plays distinct roles at different stages of the disease. Specifically, acute TWEAK activation inhibits IRI-induced cardiomyocyte death by activating the PI3K/AKT signaling pathway, thereby promoting cell survival (114). On the contrary, sustained TWEAK/Fn14 activation is detrimental and associated with chronic pathological remodeling and myocyte loss. Elevated levels of TWEAK/Fn14 contribute to chronic heart failure (HF) by promoting inflammation and fibrosis (115). Intriguingly, in a different cellular context, exposure of vascular smooth muscle cells (VSMCs) to cleaved PAI-1 (CL-PAI-1) for a long time induced apoptosis by increasing the TWEAK/FN14 pro-apoptotic signaling pathway (116). Furthermore, TWEAK is linked to the overproduction of reactive oxygen species (ROS) following IRI, which further exacerbates myocardial cell death and subsequent HF (117, 118). Overall, the pathophysiological cellular functions induced by TWEAK in CVDs are multifaceted. The dual apoptotic effect of this cytokine depends on the time point analyzed, cell type, and local environment. In the future, further exploration is needed to elucidate the potential role of TWEAK in the heart.

#### 3.2.3 TWEAK-Fn14 in angiogenesis

Angiogenesis, a complex multistep process, involves the directed proliferation and migration of endothelial cells to form new capillaries. Among the critical steps in this process, the proliferation of endothelial cells is paramount. The endothelial layer tightly regulates angiogenesis (119). TWEAK has been demonstrated to promote proliferation of both *in vitro* culture-based assays and *in vivo* models, such as rat corneal angiogenesis (120). Intriguingly, key angiogenesis regulators, including VEGFA and FGF-2, also stimulate the proliferation of endothelial cells (121, 122). Furthermore, TWEAK activates multiple signaling pathways in human endothelial cells (ECs), enhancing the mitogenic effects of FGF-2 and VEGFA, thereby promoting EC migration (123).

#### 3.2.4 TWEAK-Fn14 in cardiomyocyte hypertrophy

The most common causes of death in patients with pulmonary hypertension are associated with cardiac hypertrophy and pathological remodeling resulting from chronic pressure overload. Pathological cardiac hypertrophy is a major cause of HF (115). Recent studies have shown that overexpression of full-length TWEAK (fl-TWEAK) through gene transfection resulted in dilated cardiomyopathy (DCM) in mice, which was accompanied by a significant increase in heart weight-to-body weight ratio (HW/BW) and severe cardiac dysfunction (40). Additionally, a study have found that endogenous Fn14 was indispensable in the pathological progression of myocardial hypertrophy and that intervention in Fn14 expression attenuated pulmonary artery banding (PAB)-induced right ventricular hypertrophy (124). It was shown that TWEAK stimulation of endogenous Fn14 promotes myocyte hypertrophy via activation of the JAK2/STAT3 signaling pathway (60).

#### 3.2.5 TWEAK-Fn14 in myocardial infarction

Globally, ischemic heart disease (IHD) is a major driver of mortality and morbidity, with MI being a leading cause of death and disability (125, 126). Following experimental myocardial infarction, TWEAK and Fn14 are upregulated in cardiomyocytes. Similarly,Fn14 mRNA and protein levels increase rapidly and consistently in the left ventricle (37). Furthermore, local p38 overexpression via gene transfer upregulates Fn14 mRNA and protein levels. While the expression levels of both TWEAK and Fn14 are typically low under physiological conditions, they can be markedly upregulated under pathological conditions such as MI (23, 115). Recombinant TWEAK has been reported to increase cardiac rupture post-MI without affecting cardiomyocyte apoptosis (91). Additionally, a marked increase in TWEAK synthesis has been observed in inflammatory cells infiltrating the myocardium post-MI. Elevated serum levels of TWEAK in patients with AMI and heart failure correlate with a poor prognosis (127).

#### 3.2.6 TWEAK-Fn14 in myocardial fibrosis

Myocardial remodeling involves not only alterations in cardiac myocytes, endothelial cells, and vascular smooth muscle cells but also significant changes in interstitial cells and stroma (128). During myocardial remodeling following myocardial infarction, the expression of TWEAK and Fn14 is significantly upregulated in cardiomyocytes. Adenovirus-mediated TWEAK overexpression in mice elevates circulating sTWEAK levels, leading to dilated cardiomyopathy and subsequent severe cardiac dysfunction (40). This cardiac dysfunction results from myocardial cell elongation and the development of myocardial fibrosis. Under pathophysiological conditions, cell experiments showed that rTWEAK promotes cardiac fibrillar collagen production via TRAF3IP2 expression and activation of p38 MAPK, NF-κB, and AP-1 (42, 128–131). Specifically, NF-κB activation promotes MMP-9 release, a key mediator of myocardial hypertrophy and fibrosis. MMPs play a pivotal role in degrading the extracellular matrix, thereby influencing the dynamic balance between collagen synthesis and degradation in myocardial interstitial fibers, and are intricately linked to ventricular remodeling. The TWEAK/Fn14 axis is particularly noteworthy in its ability to promote the “aggregation” of inflammatory factors to cardiomyocytes in the early stages of myocardial infarction. Later on, it regulates extracellular matrix remodeling and fibrosis by modulating MMP-9 activity (132).

#### 3.2.7 TWEAK-Fn14 in cardiac remodeling

The pathophysiological basis of HF lies in cardiac remodeling, a complex process encompassing structural and functional alterations in cardiomyocytes, including proliferation, hypertrophy, apoptosis, necrosis, enhanced autophagy, diminished contractile function, and ventricular dilatation (133, 134). TWEAK and Fn14 are integral components of numerous biologically active processes that are intricately linked to cardiac remodeling (115, 135). Specifically, the TWEAK-Fn14 interaction facilitates cardiac remodeling by triggering the activation of the NF-κB signaling pathway. This activation subsequently leads to the induction of downstream proinflammatory chemokines such as MCP-1 and RANTES, thereby facilitating macrophage infiltration into cardiac tissue (37, 136).

In addition, PGC-1α, a key regulator of mitochondrial biogenesis, plays a pivotal role in maintaining cardiac function. Knockdown of PGC-1α leads to the downregulation of cardiac mitochondrial electron transport chain genes and a reduction in cardiac energy reserves. This, in turn, impairs cardiac performance and accelerates the progression of HF. It is postulated that the downregulation of PGC-1α contributes significantly to pathological heart injury (137–139). Moreover, the pathological mechanism of TWEAK-mediated cardiomyocyte dysfunction is closely linked to PGC-1α-dependent mitochondrial biogenesis (110). Studies have demonstrated that TWEAK reduces PGC-1α expression via both NF-κB activation and epigenetic regulation (140, 141). Similarly, experiments on muscle atrophy have revealed that the TWEAK-Fn14 interaction decreases PGC-1α levels, whereas the deletion of either TWEAK or Fn14 results in an increase in PGC-1α levels (140).

### 3.3 TWEAK-Fn14 in atherosclerosis

Atherosclerosis involves the accumulation of plaques within the arterial intima, leading to luminal narrowing, and represents a primary cause of myocardial infarction and stroke (142–144). Atherosclerosis is a multifaceted inflammatory disease encompassing various mechanisms, such as inflammation, lipid deposition, cell adhesion and smooth muscle cell proliferation, oxidative stress, and wall sclerosis, which contribute to its development (145). The interplay between TWEAK and Fn14 triggers a diverse array of biological responses, including proliferation, migration, apoptosis, angiogenesis, and the induction of inflammatory factor production, which are critically involved in various stages of atherogenesis.

#### 3.3.1 Endothelial dysfunction

Endothelial cells play a central role in the pathogenesis of atherosclerosis. As an essential part of the blood vessel wall, vascular endothelial cells are susceptible to dysfunction during the early pathophysiological stages of atherosclerosis (144). At the inception of vascular lesions, low-density lipoprotein (LDL) particles accumulate in the subendothelial space of large arteries, serving as a primary driver of endothelial cell activation (146, 147). The interaction between TWEAK-Fn14 prompts the expression of intercellular adhesion molecule-1 (ICAM-1), E-selectin, and vascular adhesion molecule (VCAM) in human umbilical vein endothelial cells (HUVECs) cultured *in vitro* (146, 147). Additionally, mTWEAK augments the expression of ICAM-1 on the surface of human brain microvascular endothelial cells (HCMECs) (101, 148). Furthermore, TWEAK stimulates the production of pro-inflammatory cytokines and chemokines in dermal microvascular endothelial cells (DMEC) (147, 149). Notably, TWEAK stimulation of HUVECs elicits the secretion of pro-inflammatory cytokines and chemokines, including IL-8 and MCP-1, which subsequently promote the migration and proliferation of smooth muscle cells (SMCs), thereby advancing plaque progression (146).

#### 3.3.2 Smooth muscle cell proliferation and migration

The initial stages of atheroma are characterized by endothelial dysfunction and monocyte adhesion, whereas the subsequent progression of atheroma involves the migration and proliferation of SMCs. Notably, marked functional differences exist between medial SMCs and neointimal SMCs within atherosclerotic plaques. As inflammatory cells infiltrate the plaque, they contribute to the phenotypic transition of SMCs towards a pro-inflammatory state, enabling them to proliferate and migrate (150). Upon stimulation with platelet-derived growth factor (PDGF) and fibroblast growth factor (FGF), SMCs migrate from the tunica media to the intimal layer, synthesizing type I and type III collagen, elastin, and glycoproteins, which collectively form the plaque matrix. Further research has demonstrated that rTWEAK plays a pivotal role in the phenotypic modulation of SMCs. Specifically, *in vitro* cell culture experiments have revealed an increased expression of bone-bridging proteins and a decreased mRNA expression of α-actinin and calmodulin in SMCs (151). These findings suggest that the TWEAK/Fn14 axis plays a crucial role in SMC proliferation and migration, contributing to intima-media thickening during the early stages of atherosclerotic plaque progression.

#### 3.3.3 Plaque instability

In atherosclerotic plaques, the ratio between the fibrous cap composed of a collagen matrix and the lipid pool is crucial for determining plaque stability. A reduction in the cellular content of the collagen matrix, coupled with an expansion of the lipid pool, can contribute significantly to plaque instability and render the plaque more prone to rupture. Matrix metalloproteinases (MMPs) acts as major mediators of fibrous cap degradation, as their increased activity weakens the fibrous structure. Notably, macrophages and smooth muscle cells (SMCs) within atherosclerotic plaques express high levels of MMPs, thereby accelerating collagen degradation and making the fibrous cap more vulnerable to rupture (152–154). The interplay between TWEAK and its receptor Fn14 is involved in promoting extracellular matrix (ECM) degradation. TWEAK and Fn14 expression is notably upregulated in macrophage-derived foam cells and VSMCs within atherosclerotic plaques, highlighting the pivotal role of these cells in the local inflammatory process (155). Specifically, TWEAK-Fn14 signaling in these cells promotes the coordinated activity of multiple collagenolytic enzymes: MMP-1 and MMP-13 primarily degrade interstitial collagens (Type I/III) of the fibrous cap, while MMP-9 targets basement membrane and denatured collagens. This synergistic action of MMPs is a critical step in reducing the tensile strength of the cap and precipitating rupture (155). Crucially, TWEAK/Fn14 interaction exacerbates plaque instability through non-MMP mechanisms as well. This axis promotes oxidative stress by activating NADPH oxidase (Nox2 and p22phox subunits) in macrophages, leading to excessive reactive oxygen species (ROS) production (156). This TWEAK-induced oxidative stress synergizes with MMP activity, further accelerating the weakening of the fibrous cap and increasing the risk of rupture (156). Furthermore, TWEAK/Fn14 signaling contributes to plaque progression by indirectly promoting the recruitment of monocytes through the induction of adhesion molecules and chemokines in vascular cells (7).Administration of rTWEAK enhances MMP activity in ApoE knockout mice, whereas treatment with an anti-TWEAK monoclonal antibody reduces MMP activity in these mice (157). These findings suggest that TWEAK plays a potentially significant role in plaque stabilization, highlighting its potential as a therapeutic target in the management of atherosclerotic plaque instability.

#### 3.3.4 Thrombus formation

Plaque rupture or erosion, followed by thrombosis, can trigger acute cardiovascular events. As atherosclerotic plaques progress to more advanced stages, endothelial dysfunction occurs, prompting endothelial cells to produce tissue factor (TF) and plasminogen activator inhibitor-1 (PAI-1). Both TF and PAI-1 are critical regulators of hemostasis and thrombosis. Specifically, TF triggers the coagulation cascade, while PAI-1 inhibits fibrinolysis. Thus, their upregulation may substantially contribute to thrombus formation following plaque rupture (158–162). The interaction between TWEAK and its receptor Fn14 has been demonstrated to upregulation the expression of PAI-1 and TF, thereby modulating thrombus formation and potentially contributing to the development of acute cardiovascular events. In human atherosclerotic plaques, Fn14 co-localizes with PAI-1 and TF, and the expression of these factors increases following rTWEAK treatment. Conversely, the application of anti-TWEAK antibodies reduces PAI-1 and TF expression in cultured SMCs (163). In summary, these findings indicate that TWEAK plays a pivotal role in atherosclerotic plaque development, spanning from its early stages to thrombus formation post-plaque rupture.

## 4 Potential role of TWEAK-Fn14 in BHS

As previously highlighted, the TWEAK-Fn14 axis occupies a pivotal position in the pathological cascades of the brain and heart. By employing drug administration or gene therapy targeting TWEAK-Fn14 signaling, we can observe a marked restoration in the functional integrity of these CCVDs. Moreover, both cerebral and cardiac injuries are capable of triggering an elevation of TWEAK levels, a phenomenon that leads to the upregulation of Fn14 expression, further perpetuating the pathological processes.

### 4.1 TWEAK-Fn14-mediated brain-heart axis as a potential mechanism for the treatment of BHS

The investigation of the intricate relationship between the brain and the heart represents a frontier and innovative research field known as neurocardiology (164, 165). Stroke patients are highly susceptible to serious adverse cardiac events. Brain injury can affect cardiac function through neural and humoral regulation. In addition, stroke can lead to injury of complex self-regulation mechanisms, leading to lowered blood flow to the brain. Cerebral blood flow is closely related to cardiac function (16, 166). However, the therapeutic targets of BHS remain poorly defined, hindering the identification of its underlying mechanisms.

Recently, BHS has attracted increasing attention. In neuromodulation, research shows that ATP released from sympathetic efferent nerves activates the NLRP3 inflammasome in the heart, triggering IL-1β production and cardiac hypertrophy (167). In addition, the ACC←VMT←DMH←Amb neuronal circuit constitute the top-down pathway of operant bradycardia that innervates parasympathetic neurons in the heart (168). Moreover, Stroke-induced atrial fibrillation occurs through the regulation of the cholinergic-calcium signaling pathway (169). Additionally, Piezo1-mediated IL-6 in the thoracic dorsal root ganglion (TDRG) is transported to the heart, thereby activating the IL-6-STAT3 pathway and exacerbating ventricular remodeling (170). In humoral regulation, studies have reported that one month after brain injury, monocytes/macrophages continue to undergo pro-inflammatory changes, and IL-1β has been confirmed to mediate innate immune-related, stroke-induced cardiac dysfunction (21). Although these studies illustrate the relationship between brain and heart from a neurohumoral perspective, they fail to elucidate how the brain affects the heart directly.

**Figure 6:**
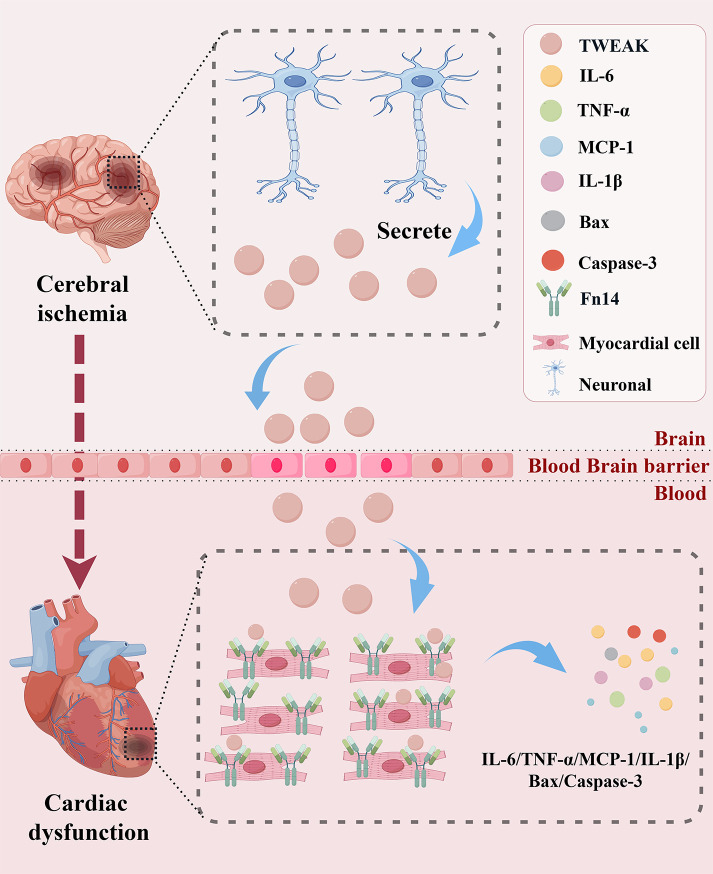
A schematic diagram of the potential mechanism of action of TWEAK-Fn14 in BHS. After ischemic stroke, brain parenchyma release a significant amount of TWEAK. This cytokine damages and penetrates the BBB into the bloodstream. Subsequently, TWEAK freely circulates to the heart, where it binds to the Fn14 receptor in cardiomyocytes. This interaction triggers the secretion of inflammatory factors, including TNF-α, IL-6, IL-1β, and MCP-1, as well as the expression of apoptotic proteins such as caspase-3 and Bax. Ultimately, these cellular changes lead to cardiac dysfunction.

As reported by our team, the TWEAK-Fn14 signaling pathway was identified as a key shared mechanism in myocardial ischemia-reperfusion injury (MIRI) and cerebral ischemia-reperfusion injury (CIRI) through RNA-seq analysis and experimental validation, elucidating the crucial role of TWEAK-Fn14 in both brain and heart (83, 171). The TWEAK-Fn14 interaction exerts multifaceted regulatory effects on cellular activities and is notably expressed in neuronal and myocardial cells (134, 135). In the brain, TWEAK secretion is notably augmented following ischemic stroke (85, 172, 173). Importantly, it can disrupt and penetrate the BBB (103, 174). In the heart, TWEAK levels in the blood are significantly elevated in patients with clinically abnormal cardiac function, suggesting its potential as a biomarker for the clinical diagnosis of cardiac dysfunction (7, 40). Transgenic or adenovirus-mediated overexpression of TWEAK leads to dilated cardiomyopathy with severe cardiac dysfunction (40). Myocardial fibrosis and cardiac dysfunction were significantly attenuated in Fn14 knockout mice (129). Fn14 protein in rat hearts significantly changes due to myocardial injury and pressure overload (175). Moreover, cell growth factor 1, norepinephrine and angiotensin II induce Fn14 expression in cardiomyocytes via the RhoA/ROCK pathway. *In vitro* studies utilizing RhoA/ROCK siRNA pretreatment effectively abolish Fn14 expression in cardiomyocytes (37). Therefore, targeting the TWEAK-Fn14 signaling pathway plays a beneficial role in protecting the heart. The TWEAK-Fn14 interaction significantly modulates the secretion of inflammatory factors, including TNF-α, IL-6, and MCP-1, and impacts the expressions of apoptotic proteins of caspase-3 and Bax (134, 176–178). In recent, our team showed that Fn14 gene was nearly seven-fold upregulated by conducting an RNA-seq analysis of the heart in a stroke-induced cardiac dysfunction model. The content of TWEAK in the brain was negatively correlated with cardiac ejection fraction (LVEF). Moreover, intracerebroventricular injection of TWEAK recombinant protein induced BBB damage and cardiac dysfunction (unpublished data). Collectively, these findings indicate that TWEAK-Fn14 axis may represent a novel mechanism underlying BHS. The possible pathological processes are as follows: TWEAK is abundantly secreted, leading to disruption and penetration of the blood–brain barrier (BBB) into the systemic circulation. TWEAK acts on the Fn14 receptor when it arrives in heart, causing cardiac dysfunction. Overall, the TWEAK-Fn14 axis provides a novel and direct humoral and immune regulatory mechanism from brain to heart for stroke-induced cardiac dysfunction (**Figure 6****).**

## 5 TWEAK-Fn14 as a clinical biomarker and target in cardio-cerebrovascular diseases

Soluble TWEAK (sTWEAK) can serve as a clinical biomarker for CCVDs, including stroke, Multiple sclerosis, carotid stenosis, myocardial infarction, coronary artery disease, peripheral arterial disease, abdominal aortic aneurysms, heart failure, diabetes, and hypertension (**Table 1****).** In stroke, a large body of experimental evidence reveals that activation of the TWEAK-Fn14 signaling pathway can lead to multiple pathological processes, thereby supporting the potential application of sTWEAK as a biomarker in brain disease. Therefore, based on these findings, the TWEAK-Fn14 axis could offer a therapeutic strategy for the pathogenesis of ischemic stroke. In CVDs, the secretion level of sTWEAK serves as a potential biomarker distinguishing normal from pathological arterial walls. For instance, lower sTWEAK levels have been linked to coronary artery disease (37, 179), systolic heart failure (37), peripheral artery disease (180), and abdominal aortic aneurysms (181, 182). Notably, sTWEAK levels have also been found to decrease in the plasma of patients with atherosclerosis (7), suggesting its potential as a biomarker for this disease. The level of TWEAK may vary in different pathological conditions, as the mechanism by which sTWEAK is reduced in patients with vascular injury may be related to the expression of its receptor. As mentioned earlier, Fn14 expression is low or undetectable in a healthy state (41). However, under pathological conditions of injury, Fn14 is highly upregulated in the vascular system, which facilitates the binding and retention of sTWEAK in pathological tissues (41, 151). Based on these findings, we speculate that the decreased concentration of sTWEAK in cardiovascular-related diseases may reflect enhanced binding to Fn14. However, this hypothesis requires validation in larger-scale studies in the future.

**Table 1:** Soluble TWEAK as a biomarker for cardio-cerebrovascular diseases in clinic.

Disease	Study type	Method	Objects	Testing samples	sTWEAK level	Function	Evidence level	Reference
Stroke	Case-Control	ELISA, RT-PCR and IHC	27	Serum	The concentration of TWEAK is significantly higher than that of the control group.	Induce neuronal apoptosis and enhance blood-brain barrier permeability.	III	(172)
Stroke	Retrospective Observational	ELISA	906	Serum	>2900 pg/mL associated with poor outcomes	Mediate glutamate excitotoxicity via BBB disruption.	III	(203)
Stroke	Prospective Case-Control	ELISA	240	Serum	Higher in deep infarcts	Correlate with WMH severity and endothelial dysfunction.	III	(204)
Myocardial infarction	Prospective Cohort	ELISA	233	Serum	The level of sTWEAK is increased.	Increase risk factors are associated with poor short-term outcomes, such as ejection fraction and infarct size.	IIb	(205)
Heart failure	Prospective Cohort	ELISA	400	plasma	The sTWEAK levels in patients with heart failure were significantly lower than those in the control group.	Predict adverse prognostic implications in patients with chronic stable heart failure.	IIb	(206)
Multiple sclerosis	Pathology/Case Series	RT-PCR and IHC analysis	12	Brain tissue	In white matter lesions of brain tissue, activated microglia can express TWEAK protein.	Induce vascular abnormalities, inflammation, astrocyte proliferation, and neuronal damage.	III	(207)
Carotid stenosis	Case-Control/Cross-sectional	Western blot and ELISA	58	Carotid plaque supernatants	The concentration of TWEAK is reduced compared to healthy individuals.	Increase carotid intima-media thickness.	III	(208)
Coronary artery disease	Prospective Cohort	ELISA	400	plasma	The sTWEAK level is significantly lower than those in the control group.	Predict the adverse prognostic implications in patients of chronic stable heart failure.	IIb	(206)
Peripheral arterial disease	Case-Control	ELISA	155	plasma	A high CD163/TWEAK plasma ratio is associated with the disease.	Reflect on the progression of atherothrombosis.	III	(180)
Abdominal aortic aneurysms	Prospective Cohort	IHC analysis and ELISA	71	plasma	The sTWEAK concentration is reduced.	sTWEAK shows an opposite trend to abdominal aortic aneurysm amplification rates.	III	(181)
Diabetes	Case-Control	ELISA	120	Serum	The sTWEAK serum level decreases in patients with type 2 diabetes mellitus (T2DM).	Cause elevated blood glucose concentration.	III	(7, 209)
Hypertension	Case-Control	ELISA	88	Serum	Young patients with primary hypertension exhibit lower levels of sTWEAK.	Decrease systolic blood pressure and diastolic blood pressure.	III	(7, 210)

The pivotal role of TWEAK-Fn14-targeted agents in alleviating brain injury and CVDs has been convincingly demonstrated in preclinical and clinical models, as outlined in **Table 2**. These therapeutic agents are broadly categorized into Fn14-Fc recombinant fusion proteins (acting as decoy receptors by capturing TWEAK) and neutralizing monoclonal antibodies (e.g., BIIB023, targeting TWEAK or Fn14). These two classes of agents represent a core clinical heterogeneity: Fn14-Fc fusion proteins are designed as soluble “decoy receptors” to sequester circulating sTWEAK, while monoclonal antibodies (mAbs) like BIIB023 provide highly specific blockade of the ligand-receptor interaction. To suppress aberrant TWEAK-dependent Fn14 signaling, further strategies are required to inhibit the pathway in injured tissues. At the preclinical level, one of the most straightforward approaches involves the use of small interfering RNAs to silence either TWEAK or Fn14 expression, thereby effectively reducing their levels (156, 183–185). Intriguingly, the employment of an anti-TWEAK neutralizing monoclonal antibody has been effective in reducing cerebral infarct size in a permanent ischemic stroke model (84). Similarly, the application of soluble Fn14-Fc decoy receptors has been reported to significantly diminish the cerebral infarct area and brain edema in a mouse stroke model (86, 100). In a notable study, it was discovered that intraperitoneal injection of recombinant soluble TWEAK and Fn14-neutralising antibodies alleviated inflammatory infiltration and disease severity in experimental autoimmune encephalomyelitis (EAE) (186).This further underscores the therapeutic potential of targeting the TWEAK-Fn14 axis in treating various pathological conditions.

**Table 2:** Undergoing clinical trials and therapeutic agents targeting TWEAK.

Study title	Method	Condition	Number of cases	Intervention	Status	Reference(s)
The effects of renin-angiotensin system blockade (RAS), calcium channel blocker, and combined drugs on TWEAK, PTX3, and FMD levels in diabetic proteinuric patients with hypertension	Randomized controlled trial (RCT)	1) Diabetes 2) Hypertension 3) Proteinuria	105	1) Drug: Amlodipine 2) Drug: Valsartan	Completed	ClinicalTrials.gov NCT00921570
Multiple sclerosis	Single-center interventional study	Multiple sclerosis	50	Diagnostic test: determination of TWEAK serum concentration	Unknown status	ClinicalTrials.gov NCT03974997
BIIB023 (Anti-TWEAK) in subjects with rheumatoid arthritis	Randomized, double-blind, placebo-controlled, single-dose, Dose-escalation study	Rheumatoid arthritis	53	1) Drug: BIIB023 2) Other: Placebo (sterile normal saline)	Completed	ClinicalTrials.gov NCT00771329
The effects of renin-angiotensin system blockage (RAS), calcium channel blocker, and combined drugs on TWEAK, PTX3, and FMD levels in diabetic proteinuric patients with hypertension	Parallel assignment	1) Diabetes 2) Hypertension 3) Proteinuria	105	1) Drug: Amlodipine 2) Drug: Valsartan	Completed	ClinicalTrials.gov NCT00921570
Does etanercept-influence TWEAK modulation of inflammation during inflammatory rheumatisms (psoriatic arthritis and rheumatoid arthritis)?	Parallel assignment	1) Inflammatory rheumatism 2) Psoriatic arthritis 3) Rheumatoid arthritis	60	Drug: Etanercept Treatment	Completed	ClinicalTrials.gov NCT02164214

The most extensively investigated TWEAK-Fn14 blockers currently in clinical trial phases are BIIB023 (NCT01943513, NCT01930890, NCT01499355, NCT00771329, and NCT01407406), RO5458640 (NCT01383733), KAHR-101 (187), BIIB036 (188, 189), and PDL192 (NCT00738764) (190, 191). Most of the clinical information about these blockers stems from the BIIB023 trials, which revealed that the downregulation of serum TWEAK levels correlated with reduced serum inflammatory biomarkers (192, 193). However, the therapeutic development of these large-molecule biologics presents significant pharmacokinetic (PK) and safety considerations (194) Specifically, the population PK analysis of BIIB023 demonstrated a multiphasic time course and a nonlinear clearance mechanism (likely due to target-mediated drug disposition, TMDD) at lower concentrations. These properties, along with a long half-life comparable to other monoclonal antibodies, support reduced dosing frequency but demand careful consideration for dosing strategies and sustained systemic exposure (195).Critically, for cerebrovascular diseases such as ischemic stroke, the efficacy of these agents is complicated by their poor blood-brain barrier (BBB) permeability (196). While acute stroke models may benefit due to BBB disruption, achieving therapeutic concentrations in the central nervous system during chronic conditions or for preventative use remains a major translational challenge, thus providing a strong rationale for the future development of small-molecule inhibitors (197). Despite these initial safety findings, the known role of the TWEAK-Fn14 axis in immune surveillance and tissue repair necessitates continuous monitoring for potential immunosuppressive adverse effects, especially in trials involving chronic dosing (198). The safety profile of BIIB023 holds significant clinical promise for its therapeutic application. In a study evaluating the human anti-TWEAK neutralizing monoclonal antibody, single-dose administration of BIIB023 demonstrated good safety and tolerability (192). The safety of different neutralizing anti-TWEAK or Fn14-Fc recombinant fusion proteins has been demonstrated, aiding in the evaluation of their therapeutic strategies. However, a more comprehensive evaluation of the safety of BIIB023 in human clinical trials is warranted in the future.

Furthermore, the pathogenesis of CCVDs suggests that TWEAK-Fn14 blockers will likely be integrated into combination strategies. Combining TWEAK inhibition (targeting inflammation and vascular remodeling) with established standard-of-care agents could offer synergistic benefits. Specific examples include co-administration with statin therapy (199) or with antiplatelet agents (e.g., aspirin, clopidogrel) (200) to simultaneously address both the inflammatory and thrombotic components of atherosclerosis and acute ischemic events.

Extracts of Ginkgo biloba (EGB) encompass a diverse array of active components that exhibit numerous pharmacological effects, including vasodilation, lipid regulation, antioxidant activity, anti-apoptosis, enhancement of cerebral blood flow, neuroprotection, and inhibition of platelet activity. These properties of EGB are useful in the treatment of CCVDs, Alzheimer’s disease, atherosclerosis, cancer, asthma, non-alcoholic fatty liver disease, diabetes, and their associated complications (201, 202). The primary constituents of EGB are ginkgolides and ginkgo flavonoids. Studies have demonstrated that EGB can reduce the secretion of TWEAK and the expression of the Fn14 protein, thereby modulating the TWEAK-Fn14 pathway to mitigate cerebral and myocardial ischemic injury in mice (83, 171). Exploring the mechanism of EGB’s action in treating BHS induced by ischemic stroke from a multidimensional perspective holds significant scientific value.

## 6 Discussion

In this review, we provide a systematic summary of the structure, function and pathway, as well as the diverse roles of TWEAK-Fn14 in CCVDs, atherosclerosis, and BHS. TWEAK-Fn14 axis contributes to BBB damage, brain edema, neuroinflammation, neuronal apoptosis, and neurodegeneration in brain, while is involved in cardiomyocyte proliferation, inflammation, apoptosis, hypertrophy, fibrosis, contractile function disruption, and ventricular dilatation in heart. Based on our descriptions, the TWEAK-Fn14 axis represents the direct mechanistic link in SHS elucidating the cascade from brain-derived TWEAK secretion. TWEAK can damage and penetrate the BBB, enter into the humoral circulation, and subsequently bind to the Fn14 receptor in cardiomyocytes, resulting in cardiac dysfunction. Moreover, the bidirectional regulation of TWEAK-Fn14 in CCVDs depends on the disease stage, and various cell types, distinct molecular mechanisms and functions need to be further studied. The BHS concept resonates with the holistic view of traditional Chinese medicine (TCM), advocating for the same treatment of diverse diseases. Emerging evidence further suggests that key components of these TCM formulations may directly or indirectly modulate the TWEAK-Fn14 axis. For example, Shuxuening injection (SXNI) and its active components, such as ginkgolides and flavonoids, have been shown to modulate TWEAK-Fn14 signaling in models of cerebral ischemia and myocardial injury. Specifically, ginkgo flavonoids downregulate TWEAK and Fn14 expression in myocardial ischemia-reperfusion injury, while ginkgolides upregulate these components in cerebral ischemia-reperfusion models, indicating context-specific regulation (83). These mechanistic features suggest that other TCM formulations containing Ginkgo biloba, including Shuxuening injection, Ginkgo Leaves Capsule and Yindan Xinnaotong capsule, may also modulate the TWEAK-Fn14 axis and provide coordinated cardio-cerebral protection.

However, despite the appeal of the TWEAK-Fn14 axis as a therapeutic target for BHS, its translation into clinical practice must confront the limitations revealed by current clinical trials. First, target specificity remains an unresolved concern because TWEAK is widely expressed and participates in both pathological and physiological tissue-repair processes, raising the risk of off-target modulation and impaired regeneration (53, 76, 211–213). Second, safety and long-term tolerability have yet to be fully characterized, as existing data are largely derived from short-term and small-sample studies, with limited information on chronic administration or combination therapy effects (192, 214). Third, inter-individual variability in TWEAK-Fn14 expression, potentially influenced by genetic polymorphisms, inflammatory microenvironment, or disease stage, may lead to heterogeneous treatment responses, thereby complicating the identification of suitable patient subgroups. Beyond these clinical hurdles, several limitations in foundational research must also be addressed before the TWEAK-Fn14 axis can be translated into therapeutic strategies.

Reliance on animal models warrants caution because species differences in cardiomyocyte biology and inflammatory responses may limit translational relevance (115, 215). Second, the current spectrum of BHS models is predominantly centered on stroke-induced cardiac dysfunction, leaving other potential triggers (e.g., epileptic or stress-induced cardiac damage) largely unexplored (216, 217). Third, the intricate cross-regulation between the TWEAK-Fn14 network and other cytokine pathways (e.g., TNF-α, IL-6) remains poorly defined. This obscures the precise mechanistic contribution of TWEAK-Fn14 within the multifactorial pathophysiology of BHS. Finally, prospective clinical validation of soluble TWEAK (sTWEAK) as a BHS biomarker is still lacking. Optimal detection windows and diagnostic cut-off values remain undefined.

To overcome these bottlenecks, future research should leverage advanced technologies in a targeted manner. The application of single-cell RNA-sequencing to human post-mortem brain-heart tissues could precisely map cell-type-specific TWEAK-Fn14 expression, clarifying its human relevance (218). Modular organ-on-a-chip systems reconstituting the neurovascular unit and myocardial tissue could experimentally model TWEAK trafficking and target engagement, empirically validating the humoral hypothesis. Furthermore, large-scale, multicenter prospective cohorts are essential to establish clinically actionable sTWEAK kinetic profiles and threshold concentrations, ultimately translating this biomarker into diagnostic and prognostic algorithms for BHS. Targeting the TWEAK-Fn14 axis may represent a hematological and inflammatory regulatory strategy for BHS management. Post-stroke cardiac dysfunction is multifaceted, involving the HPA axis activation, autonomic imbalance, gut-microbiota dysbiosis, immune dysregulation, and circadian disruption, all of which merit future attention (11, 16, 17, 219, 220). Contemporary methodologies, including Mendelian randomization, multi-omics, chemoproteomics, organoids-on-chip, and spatial mass spectrometry imaging, offer promising avenues for delving into the complexities of BHS. We anticipate that collaborative efforts between cardiologists and neurologists, supported by these advanced methodologies, will deepen our understanding of BHS pathophysiology and accelerate translational progress.
